# Role of Fibroblast Growth Factor-23 in Innate Immune Responses

**DOI:** 10.3389/fendo.2018.00320

**Published:** 2018-06-12

**Authors:** Elizabeth A. Fitzpatrick, Xiaobin Han, Zhousheng Xiao, L. Darryl Quarles

**Affiliations:** ^1^Department of Microbiology, Immunology and Biochemistry, University of Tennessee Health Science Center, Memphis, TN, United States; ^2^Department of Medicine, University of Tennessee Health Science Center, Memphis, TN, United States

**Keywords:** fibroblast growth factor-23, innate immunity, klotho, macrophage activation, PMNs, LPS stimulation, infection risk

## Abstract

Fibroblast growth factor-23 (FGF-23) is a bone-derived hormone that activates FGFR/α-Klotho binary complexes in the kidney renal tubules to regulate phosphate reabsorption and vitamin D metabolism. The objective of this review is to discuss the emerging data that show that FGF-23 has functions beyond regulation of mineral metabolism, including roles in innate immune and hemodynamic responses. Excess FGF-23 is associated with inflammation and adverse infectious outcomes, as well as increased morbidity and mortality, particularly in patients with chronic kidney disease. Enhancer elements in the FGF-23 promoter have been identified that mediate the effects of inflammatory cytokines to stimulate FGF-23 gene transcription in bone. In addition, inflammation induces ectopic expression of FGF-23 and α-Klotho in macrophages that do not normally express FGF-23 or its binary receptor complexes. These observations suggest that FGF-23 may play an important role in regulating innate immunity through multiple potential mechanisms. Circulating FGF-23 acts as a counter-regulatory hormone to suppress 1,25D production in the proximal tubule of the kidney. Since vitamin D deficiency may predispose infectious and cardiovascular diseases, FGF-23 effects on innate immune responses may be due to suppression of 1,25D production. Alternatively, systemic and locally produced FGF-23 may modulate immune functions through direct interactions with myeloid cells, including macrophages and polymorphonuclear leukocytes to impair immune cell functions. Short-acting small molecules that reversibly inhibit FGF-23 offer the potential to block pro-inflammatory and cardiotoxic effects of FGF-23 with less side effects compared with FGF-23 blocking antibodies that have the potential to cause hyperphosphatemia and soft tissue calcifications in animal models. In conclusion, there are several mechanisms by which FGF-23 impacts the innate immune system and further investigation is critical for the development of therapies to treat diseases associated with elevated FGF-23.

Fibroblast growth factor-23 (FGF-23) is a bone-derived hormone that participates in a bone–kidney endocrine network regulating mineral metabolism. FGF-23 is produced by osteoblasts and osteocytes in bone and enters the circulation to inhibit phosphate reabsorption and reduce serum 1,25D levels through the activation of canonical FGFR/α-Klotho receptor complexes in renal tubules (1–3). The major physiological functions of FGF-23 are to (1) coordinate bone mineralization with renal handling of phosphate (1, 4) and (2) act as a counter-regulatory hormone for 1,25D (Figure 1A). Elevated levels of FGF-23 are associated with several hereditary and acquired hypophosphatemic disorders including X-linked hypophosphatemic rickets (Hyp mice homolog)—caused by inactivating mutations of *Phex* (5–7)—autosomal recessive hypophosphatemic rickets 1—caused by inactivating mutations of *Dmp1* (5, 7)—ARHR2—caused by inactivating mutations in *Enpp1* (6–10)—and Raine syndrome—caused by inactivating mutations in FAM20C (11, 12). Increased FGF-23 is the result of either increased gene transcription or diminished cleavage of FGF-23 in these disorders.

**Figure 1:**
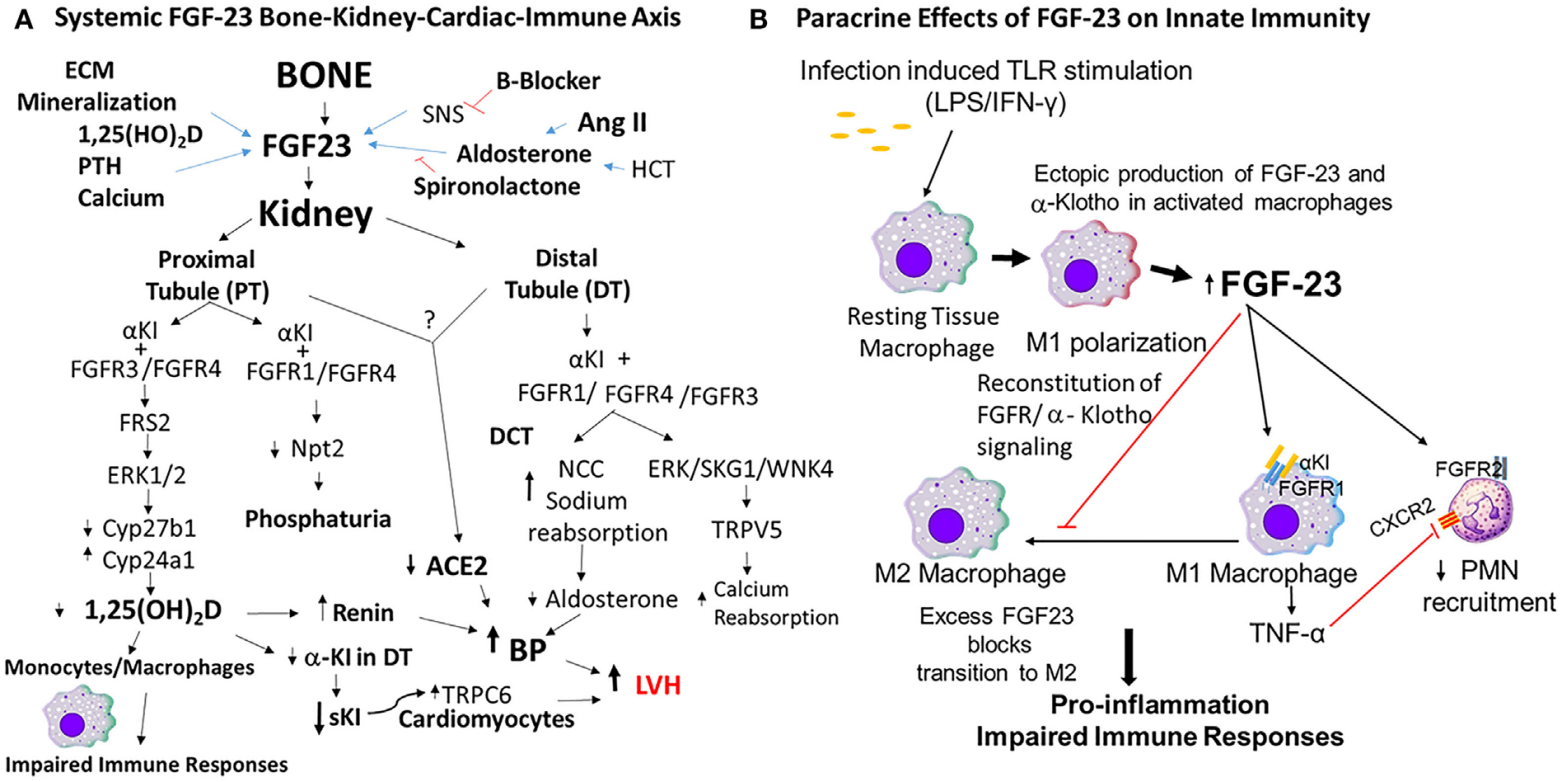
Mechanisms of fibroblast growth factor-23 (FGF-23) effects to impair innate immune responses. **(A)** FGF-23 bone–kidney–cardiac–immune axis. FGF-23 is produced by osteoblasts/osteocytes in bone in response to local and systemic factors and targets the kidney to create multiple endocrine networks (4, 13) in bone, including (1) an FGF-23 vitamin D counter-regulatory loop (4, 14); (2) a calcium-FGF-23 endocrine loop, where calcium stimulates FGF-23 in bone and FGF-23 stimulates calcium reabsorption in the DT (15, 16); (3) a bone–kidney axis, where FGF-23 is regulated by factors involved in extracellular matrix mineralization to coordinate renal phosphate handling (1). In addition, there is a bone–renal–cardiac axis that augments hemodynamic responses through a feed forward bone–cardio–renal loop, where angiotensin II (Ang II) stimulates FGF-23 production in bone (17) and FGF-23 suppresses Ace2 in the kidney (18), a volume regulatory loop where diuretics and aldosterone stimulate FGF-23 production by bone and FGF-23 targets the distal tubule to increase sodium reabsorption, and a FGF-23 soluble Klotho (sKl) regulatory axis, where FGF-23 suppresses α-Kl expression in the distal tubule leading to reduction in sKl and loss of the hormonal effects of this antiaging hormone. In this schema, FGF-23 regulates innate immune response through suppression of 1,25(OH)D2 production by the kidney. **(B)** Paracrine effects of FGF-23 on innate immune responses. Macrophages do not normally express FGF-23 or α-Klotho, but in the setting of infection, LPS/IFNγ induces the ectopic expression of both FGF-23 and its co-receptor α-Klotho that reconstitutes paracrine FGF-23 signaling in macrophages. FGF-23 stimulates pro-inflammatory responses in M1 macrophages and blocks the transition to M2 macrophages (3). In addition, FGF-23 is proposed to directly activate FGFR2 in PMNs to decrease recruitment (19).

## Role of FGF-23 in Inflammation

There are emerging data that FGF-23 may have effects on immune responses. Vitamin D, which is regulated by FGF-23, has well-described effects on both innate and adaptive immunity (20–22). Vitamin D has an overall effect to enhance innate immune responses and exert anti-inflammatory effects through local and systemic effects. Evidence that FGF-23 may have an overall impact on immunity comes from the association between elevated FGF-23 levels and inflammation. FGF-23 is increased in inflammatory bowel disease (23) and chronic kidney disease (CKD) (4). In CKD, elevated FGF-23 initially functions to maintain mineral homeostasis, but persistent elevations are maladaptive and associated with increased morbidity and mortality (24–26), cardiovascular disease (26–30), inflammation, and infections (31, 32). Infections, most commonly caused by infected catheters and pneumonia (33), are second to cardiovascular disease in causing death in CKD (34) and are >100-fold higher than the general population. Recent clinical association studies suggest that elevated FGF-23 contributes to an increase in susceptibility or severity of infections in CKD. Moreover, elevated FGF-23 levels correlated with increased IL-6, TNFα, CRP, fibrinogen, and severe inflammation in CKD patients (31, 35). In a CKD mouse model of bacterial pneumonia, FGF-23 administration exacerbated disease severity and its inhibition improved outcomes (19). Although these studies suggest that FGF-23 interacts with the immune system, they do not reveal whether FGF-23 directly regulates immune cell functions or indirectly affects immune responses through FGF-23 regulation of 1,25D.

## Inflammatory Stimuli Induce FGF-23 Expression in Osteoblasts/Osteocytes and Immune Cells

Inflammatory stimuli upregulate FGF-23 expression in bone, where FGF-23 is usually expressed, but also in immune cells and tissues that do not normally express FGF-23. Inflammatory cytokines have been shown to induce FGF-23 expression *in vivo* and *in vitro*. Inflammation increases circulating FGF-23 levels in both animal models and humans in response to infection, inflammation, and oxidative stress (36–38). For example, mouse serum FGF-23 levels are significantly increased following inoculation with Gram-negative (*E. coli*) or Gram-positive (*S. aureus*) bacteria, or LPS administration in mice (37). Numerous studies have demonstrated that FGF-23 transcription in osteoblasts/osteocytes is regulated by LPS, IL-1β, and TNFα (39). An enhancer residing in the −16 kb region of FGF-23 was identified and following deletion using CRISPR/Cas9 technology was demonstrated to be responsible for the stimulation of FGF-23 transcription by inflammatory stimuli (40). Bacterial components stimulate immune cells through toll-like receptors (TLRs) and stimulation of TLRs 2, and 4 on bone marrow-derived dendritic cells increased FGF-23 mRNA expression. TLR4 recognizes the bacterial cell wall component LPS, and TLR2 recognizes lipotechoic acids present in bacterial membranes leading to the activation of NF-κB pathway. Inhibition of NF-κB activation prevented the upregulation of FGF-23 mRNA in response to LPS stimulation. Hif1α is another transcription factor that stimulates FGF-23 in osteoblasts (41), indicating that oxidative stress/inflammation stress may induce expression of FGF-23.

Macrophages may be central to FGF-23 regulation and function. Resting RAW264.7 macrophages, which do not normally express FGF-23, when stimulated with LPS/IFN-γ to induce M1 polarization express significant increases in FGF-23. The increase is also mediated by NF-κB-dependent activation of the FGF-23 promoter (3). In addition, the M1 macrophages upregulate Klotho transcription, including the full-length α-Kl message and the alternatively spliced s-Kl message and protein levels. By contrast, IL-4 induction of M2 macrophages results in only modest increases in FGF-23 expression and no upregulation of α-Klotho transcription. Not only do macrophages make FGF-23 and respond to FGF-23 (see below), but make other cytokines, such as OSM, that induce tissue expression of FGF-23, such as in cardiomyocytes (42). In contrast to M1 macrophages, Masuda et al. (37) did not observe upregulation of FGF-23 mRNA in T cells stimulated with the polyclonal stimulators PMA and ionomycin. Lymphocytes also express TLRs 2 and 4, and it is not known whether stimulation through these receptors will upregulate FGF-23 mRNA in these cells.

These observations suggest that inflammatory mediators stimulate FGF-23 gene transcription in osteoblasts/osteocytes that normally express FGF-23 and in macrophages and tissues that do not normally express FGF-23. The resulting local and systemic production of FGF-23 may play a critical role in the ensuing immune responses through targeting FGFR/α-Klotho receptor complexes in the kidney or reconstituted receptor complexes in the local inflammatory tissue environment.

## Potential Mechanisms for FGF-23 Impairment of Host Responses

### Indirect Effects Mediated by FGF-23 Suppression of 1,25D

Under physiological conditions, changes in FGF-23 results in reciprocal changes in 1,25D, and *vice versa*. FGF-23 may affect immune responses through activation of FGFR/α-Klotho complexes in the proximal renal tubule leading to suppression of 1,25D by the kidney (3, 19, 43–46). The association between low vitamin D and high FGF-23 serum levels with infectious and cardiac deaths in a large cohort of patients with end-stage renal disease (47, 48) may be due to the loss of the anti-inflammatory effects of 1,25D (49, 50). The loss of 1,25D may result in the amplification of inflammatory response and subsequently increased tissue pathology.

### Direct Effects of FGF-23 on Macrophages and PMNs

Another mechanism is direct pro-inflammatory actions of FGF-23 on immune cells. There is emerging evidence that FGF-23 directly interacts with immune cells, such as PMNs and/or macrophages through binding of FGFR/α-Kl receptors (Figure 1B).

Rossaint et al. (19) used a murine model of CKD, induced by 5/6 nephrectomy, to measure the effect of excess FGF23 on *E. coli* pneumonia. The authors demonstrate that mice with CKD had decreased recruitment of PMNs into the lungs and increased disease severity. Neutralization of FGF23 with anti-FGF23 antibody restored PMN recruitment and the host response in these mice. The authors proposed that elevated circulating levels of FGF-23 directly activated FGFR2 in PMNs to impair the host response to infection.

Direct effects of FGF-23 on PMNs are controversial for several reasons. First, PMNs lack the obligate FGF-23 co-receptor α-Klotho. To deal with this inconsistency, this hypothesis proposes that FGF-23 targets PMNs through the non-canonical FGFR2 pathway (2). Second, FGFR2, which is the only FGFR expressed in PMNs, is not a target for FGF-23 in multiple functional studies and target engagement assays (2, 51–53). Third, experimental designs in existing studies are not sufficient to establish the role of FGFR2 or PMNs in mediating FGF-23’s adverse effects on host responses. In this regard, a single intravenous injection of 200 ng of rFGF-23 to mice was the only *in vivo* evidence that elevated FGF-23 impairs PMN responses to sepsis. Essential studies to ablate FGFR2 in PMNs or to test the effects of chronic elevations of FGF-23 in the absence of confounding effects of CKD (19, 54) have not been performed. Finally, effects of FGF-23 inhibition to improve outcomes with a blocking antibody have not controlled for confounding effects of reciprocal increases in 1,25D production in response to FGF-23 inhibition.

There is compelling data that FGF-23 targets macrophages. Several studies have demonstrated that macrophages and DCs express FGFR1 and inflammatory stimuli upregulates α-Klotho expression in macrophages to reconstitute FGFR–α-Klotho signaling (3, 37). Stimulation of these cells with FGF-23 resulted in induction of TNF-α mRNA and protein expression in primary macrophages as well as macrophage cell lines through activation of binary FGFR/α-Klotho complexes (3, 37). In addition, the ability of FGFR inhibitors to block FGF-23 signaling in macrophages confirms that the canonical FGFR/Kl signaling pathway is active in macrophages (3).

If FGF-23 is directly affecting innate immune responses, animal models with elevated FGF-23 should exhibit abnormal host responses even in the absence of CKD. Indeed, a sterile inflammation model to induce the infiltration of peritoneal macrophages with thioglycolate shows that Hyp mice have increased FGF-23 expression in macrophages (3). Macrophages isolated from Hyp mice expressed higher levels of FGF-23 and Klotho compared with WT controls. Hyp macrophages also had increased basal ERK activation and increased TNF-α mRNA, consistent with activation of FGFR/αKl signaling. Serum levels of TNF-α were increased consistent with the pro-inflammatory phenotype [i.e., kidney inflammation (18) and cardiovascular abnormalities in Hyp mice (55)]. These data support the hypothesis that locally produced and circulating FGF-23 activate immune cells in the inflammatory milieu (3, 43), leading to adverse outcomes (56). In this scenario, infection with bacteria stimulates the production of FGF-23 locally in M1 activated macrophages which also upregulate FGFR/α-Kl complexes (3). Autocrine stimulation of the M1 cells with FGF23 amplifies TNF production and inhibits the transition to a wound healing M2 phenotype, resulting in excess tissue damage and increased morbidity and mortality (Figure 1B).

Based on these findings, a new hypothesis proposes that FGF-23 actions on innate immunity are mediated by activation of reconstituted canonical FGFR/α-Kl receptors in tissue macrophages during infections by both systemic and local production of FGF-23 (Figure 1B) (3, 19, 37, 43). Although the full effects of FGF-23 on immune cell function remain to be defined, in this schema, FGF-23 is proposed to have pro-inflammatory effects mediated by activated macrophages. Additional studies are needed to characterize the impact of FGF-23 on immune responses. In particular, experiments that conditional delete FGF-23, FGFRs, and α-Klotho in different myeloid cells are needed to define the role of FGF-23/FGFR/α-Klotho signaling in mediating immune responses.

## Role of α-Klotho and Soluble Klotho (sKl) in Inflammation

α-Klotho gene has two transcripts that encode a long type I transmembrane (TM) protein containing KL1 and KL2 domains and a short secreted protein containing only a single KL domain (57). The ~130-kDa TM protein is an obligate co-receptor for binding of FGF-23 to FGFRs (2, 58). Ectodomain shedding by ADAM10 and ADAM17 generates a circulating α-Klotho isoform that lacks the TM domain (59). The short secreted ~60-kDa isoform (s-KL) is generated by the alternative spliced transcript. Similar sized as soluble KL1 and KL2 fragments are also generated by additional post-translational cleavage of the shed isoform (57, 60). The ~60-kDa s-KL gene product emerged during evolution before FGF-23 and likely has FGF-23 independent functions, including antiaging, anti-inflammatory, and anti-fibrotic effects due to actions of secreted forms of KL to inhibit Wnt, IGF-1, and TGF-β signaling (61–65). Since excess FGF-23 is associated with decreased expression of α-Klotho in the kidney and circulating Kl, it is difficult to determine if the adverse effects attributed to excess FGF-23 are actually caused by Klotho deficiency. Indeed, Klotho depletion is associated with increased inflammation in multiple experimental models.

A recent study showed that renal Klotho mRNA and protein were significantly decreased leading to increased inflammation in kidney of the *db/db* mouse model of diabetes (66). Addition of sKl or overexpression of α-Klotho suppressed NF-κB activation and subsequent production of inflammatory cytokines in response to TNF-α stimulation *in vitro*. Klotho serves as an anti-inflammatory modulator, which negatively regulates the production of NF-κB-linked inflammatory proteins *via* a mechanism that involves phosphorylation of Ser^536^ in the transactivation domain of RelA (66). Thus, distinguishing between FGF-23 and α-Klotho-dependent effects are important, because Klotho administration would correct abnormalities caused by FGF-23 suppression of Klotho.

## FGF-23 Regulates Hemodynamic Responses to Counter the Hypotensive Effects of Inflammation

Could FGF-23 effects regulate renal process that have cardiovascular effects, be linked to inflammatory responses (53, 67)? For example, FGF-23 upregulates NCC in the DT leading to hypertension and suppression of aldosterone levels (15, 16, 55). FGF-23 administration suppresses ACE2 and αKl expression in the kidney, which could potentially cause left ventricular hypertrophy by enhancing the response to Ang II and/or decreasing circulating sKl (68). From a teleological perspective, FGF-23 control of blood pressure may serve to attenuate the hypotensive effects of inflammation, or alternatively account for the link between inflammation and hypertension (69).

## Therapeutic Potential of FGF-23 Antagonist

If elevations of FGF-23 are causally linked to adverse outcomes, as emerging data are suggesting, then efforts to inhibit the end-organ effects of excess FGF-23 may improve outcomes in clinical conditions of FGF-23 excess. Advancements in pharmacological tools to block FGF-23 have been made, including blocking antibodies, which show efficacy and safety in treating hypophosphatemic rickets in hereditary disorders of FGF-23 excess (70). Blocking antibodies, however, have a narrow safety window and long half-life (16, 70), which may limit their use in CKD. Indeed, FGF-23-blocking antibody has not been shown to safely reduce FGF-23 in CKD without worsening hyperphosphatemia. Use of an FGF-23-blocking antibody also induced aortic calcification associated with increased risk of mortality in a rodent model of CKD (16). A novel FGF-23 antagonist, Zinc13407541, has been developed, which is short-acting and effectively antagonizes FGF-23 actions *in vitro* and *in vivo* without elevating serum phosphate (71). Unlike FGF-23-blocking antibodies, this small molecule compound can specifically block FGF-23/FGFR/Klotho signaling and increase serum 1,25(OH)_2_D levels (71) and suppresses TNF-α expression in activated macrophages. With further lead optimization, derivatives of Zinc13407541 could potentially be a titratable pharmacological tool to block FGF-23-related mortality in CKD.

In conclusion, a new understanding of FGF-23 functions proposes functions beyond mineral metabolism that includes indirect and direct effects on components of the myeloid lineage that may account for the association between elevated FGF-23 and impaired host responses to infections. Distinguishing between the proposed mechanisms, namely, FGF-23 suppression of 1,25D, activation of FGFR2 in PMNs in the absence of α-Klotho, and FGF-23 activation of canonical FGFR/α-Kl signaling in macrophages, is important, if we are to make progress in preventing and treating complications of excess FGF-23. Additional studies are needed to determine the relative importance of FGF-23 direct effect on different components of the myeloid lineage in regulating innate immune function and the clinical significance of FGF-23 actions to counteract the systemic and local immune effects of Vitamin D. Distinguishing between these direct and indirect effects will help establish whether pharmacological inhibition of FGF-23 or administration of 1,25D or sKl can prevent the adverse outcomes associated with FGF-23 excess.

## Author Contributions

XH performed the *in vitro* studies characterizing the ectopic expression of FGF-23 in activated macrophages. EF performed investigations evaluating impact of FGF-23 on the susceptibility to infection. ZX performed the studies characterizing regulation of FGF-23. LQ supervised all research studies related to FGF-23 effects in macrophages. All the authors contributed to the literature review and writing of this manuscript.

## Conflict of Interest Statement

The authors declare that the research was conducted in the absence of any commercial or financial relationships that could be construed as a potential conflict of interest.
